# Evaluation of endogenous control gene(s) for gene expression studies in human blood exposed to ^60^Co γ-rays *ex vivo*

**DOI:** 10.1093/jrr/rru074

**Published:** 2014-09-30

**Authors:** S. Thangminlal Vaiphei, Joshua Keppen, Saibadaiahun Nongrum, R.C. Chaubey, L. Kma, R.N. Sharan

**Affiliations:** 1 Radiation and Molecular Biology Unit, Department of Biochemistry, North-Eastern Hill University (NEHU), Shillong, 793022, India; 2 Radiation Biology and Health Sciences Division, Bhabha Atomic Research Centre (BARC), Trombay, Mumbai, 400085, India; 3 Radiation Countermeasures Unit, Department of Biochemistry, North-Eastern Hill University (NEHU), Shillong, 793022, India

**Keywords:** human peripheral blood lymphocyte (HPBL), gene expression, qPCR, endogenous control gene, ^60^Co γ-rays

## Abstract

In gene expression studies, it is critical to normalize data using a stably expressed endogenous control gene in order to obtain accurate and reliable results. However, we currently do not have a universally applied endogenous control gene for normalization of data for gene expression studies, particularly those involving ^60^Co γ-ray-exposed human blood samples. In this study, a comparative assessment of the gene expression of six widely used housekeeping endogenous control genes, namely *18S*, *ACTB*, *B2M*, *GAPDH*, *MT-ATP6* and *CDKN1A*, was undertaken for a range of ^60^Co γ-ray doses (0.5, 1.0, 2.0 and 4.0 Gy) at 8.4 Gy min^−1^ at 0 and 24 h post-irradiation time intervals. Using the NormFinder algorithm, real-time PCR data obtained from six individuals (three males and three females) were analyzed with respect to the threshold cycle (Ct) value and abundance, ΔCt pair-wise comparison, intra- and inter-group variability assessments, etc. *GAPDH*, either alone or in combination with *18S*, was found to be the most suitable endogenous control gene and should be used in gene expression studies, especially those involving qPCR of γ-ray-exposed human blood samples.

## INTRODUCTION

In the post-genomic era and the shifting paradigms of radiation biology, studies in the domain of molecular radiobiology involve assessment of gene expression following irradiation by techniques such as Quantitative Real-Time PCR (qPCR), DNA Microarray, etc. Such assessments require a complex mathematical algorithm involving an endogenous control gene [1–11]. An ideal endogenous control gene ought to be constitutively expressed and invariant for a range of experimental conditions and interventions, subjects, tissues, etc. A housekeeping gene meets these criteria and, hence, is normally used as the endogenous control gene to normalize background gene expression levels. A glance through the published literature shows that a range of endogenous control, reference or normalizer genes have been used in various studies. It is obvious that the different reference genes would vary in their native and induced expressions in response to treatments or experimental conditions, as well as between subjects, tissues, etc. [3, 9, 11–20]. For these reasons, combining the results of the various studies (and interlab comparison of results) is difficult. An inappropriate reference or control gene may also lead to misinterpretation of the gene expression data. However, to date there is no consensus on a universal endogenous control gene. Thus, there is an urgent need to standardize the procedure by finding one or two of the most suitable endogenous control genes (by consensus) that exhibit minimal variation in gene expression results and permit comparison of the findings in the various studies and laboratories.

Some attempts have been made in the past to identify stable and convenient endogenous control genes in human studies [10, 16, 21]. The *18S* and *β-actin* genes have been used in irradiated human blood as reference genes for normalization [21–24]. In some studies, *PPIB* [16] and a combination of the *TRAP1*, *FPGS*, *DECR1* and *PPIB* [10] genes have been used as reference genes in studies involving human peripheral blood. In other studies, while the *GAPDH*, *B2M* and *ACTB* genes were shown to be reliable reference genes in peripheral blood mononuclear cells in post-traumatic stress disorder patients [25], *β-actin* and *TUBB1* were used as the reference genes in human skin fibroblasts after UVB irradiation [26]. Similarly, the *18S* gene alone was used as a normalizer gene in irradiated human fibroblasts [27]. On the other hand, many reports show that the reference genes used were not stable. For example, low-dose X-ray irradiation was reported to downregulate *β-actin* up to 17 h post-radiation in human peripheral blood lymphocytes *in vitro* [28]. The expression of the *CDKN1A* gene was also demonstrated to be upregulated in the blood of patients undergoing total body irradiation [22]. While the *18S* and *B2M* genes were reported to be unstable under different radiation qualities in two human cell lines, the *GAPDH* and *ATP6* genes were reportedly stable and, hence, were used as the reference genes [29]. For obvious reasons, unstable genes do not make good normalizer genes. As a consequence of this, gene expression analysis using qPCR also utilized normalization to intergenic and intragenic regions of candidate radiation-responsive genes for dose prediction as well as reduced interindividual variations in the absence of untreated basal gene expression [30]. Hence, it is apparent that there is currently no universal reference gene that is stably and abundantly expressed under various experimental conditions and able to serve as an ideal and common endogenous control gene [2, 20, 31].

To the best of our knowledge and belief, so far no comparative assessment has been made between the commonly used endogenous control genes in human blood exposed to ^60^Co γ-rays in order to find the most suitable normalizer gene for gene expression studies. Therefore, the primary goal of this study was to make a comparative analysis of the commonly used endogenous control genes for their suitability to use in gene expression studies. To achieve this goal, we have examined six housekeeping genes, namely *18S* (ribosomal protein), *ACTB* (β-actin), *B2M* (β-2-microglobulin), *GAPDH* (glyceraldehyde-3-phosphate dehydrogenase), *MT-ATP6* (mitochondrially encoded ATP synthase 6) and *CDKN1A* (cyclin-dependent kinase inhibitor 1A), in human whole blood by qPCR, either immediately (0 h group) or at 24 h post-irradiation period following exposure to a range of doses of ^60^Co γ-rays.

## MATERIALS AND METHODS

### Sample preparation

Approximately 5 ml of blood was collected from each of six consenting volunteers (three males and three females) in the age range of 25–30 years. Each sample was distributed into five equal parts in 1.5 ml Eppendorf tubes. Tube 1 served as the sham-exposed control, while tubes 2 to 5 were exposed *ex vivo* to ^60^Co γ-rays at 0.5 Gy, 1.0 Gy, 2.0 Gy and 4.0 Gy, respectively (LDBI 2000 purchased from BRIT, BARC, Mumbai; dose rate of ∼8.39 Gy min^−1^). Equal volumes (0.25 ml each) of sham-exposed and irradiated samples were aliquoted into two new tubes. To each of these tubes, 0.25 ml RPMI-1640 (Sigma–Aldrich) medium supplemented with 10% heat-inactivated fetal bovine serum (Hi-Media) was added [23]. Since the post-irradiation incubation periods of 0 (immediately after irradiation) and 24 h were chosen for the two sets of samples, 0.75 ml of TRI Reagent BD (Sigma–Aldrich) and 25 μl of 5N acetic acid were added immediately in one set (0 h post-irradiation group), then mixed and stored at −50°C. The second set (24 h post-irradiation group) was transferred to a CO_2_ incubator (Thermo Fisher). After 24 h in the CO_2_ incubator (15% CO_2_; ∼100% humidity; 37°C), equal volume of TRI Reagent BD and 5N acetic acid were added and mixed as before. The samples were stored at −50°C until further use.

### RNA isolation and cDNA synthesis

Total RNA was isolated directly from the frozen whole blood–TRI Reagent BD mixture according to the manufacturer's (Sigma–Aldrich's) instructions. RNA concentration and purity were estimated using a NanoDrop 2000c (Thermo Fischer), and A_260/A280_ values of >1.8 were considered to be satisfactory. For cDNA synthesis, a High Capacity cDNA Reverse Transcription Kit (Applied Biosystems) was employed with 1.0 µg of the RNA template and random hexamer primers, according to the manufacturer's instructions. The conditions of reactions in the thermo cycler were 25°C for 10 min, 37°C for 120 min and 85°C for 5 min. In order to check for genomic DNA contamination, reactions without Reverse Transcriptase (RT) were also run to serve as ‘−RT’ controls. Primers containing two exon boundaries were also employed to avoid genomic DNA contamination. The cDNA samples were stored at −50°C until further use.

### qPCR analysis

For gene expression analysis of all six housekeeping genes (*18S*, *ACTB*, *B2M*, *GAPDH*, *MT-ATP6* and *CDKN1A*), TaqMan Gene Expression Assays (Applied Biosystems) were employed (Table 1). The qPCR was carried out with an Applied Biosystems 7500 Fast Real-Time PCR system with 5 µl TaqMan Fast Universal PCR Master Mix in a 10-µl reaction volume. The optimized thermal cycling conditions in Fast Mode were 95°C for 2 min, 40 cycles at 95°C for 3 s each and 60°C for 30 s.

**Table 1.** List of endogenous genes selected for this study

Gene symbol	Gene name	Function	Assay ID
*18S*	*Eukaryotic 18S ribosomal RNA*	Component of ribosomal subunit (40S)	Hs99999901_s1
*ACTB*	*Beta-actin*	Cell motility, structure and integrity	Hs99999903_m1
*B2M*	*Beta 2-microglobulin*	Component of MHC I on all nucleated cells, protein binding, antigen presentation	Hs99999907_m1
*GAPDH*	*Glyceraldehyde-3-phosphate dehydrogenase*	Glycolytic enzyme involved in the breakdown of glucose	Hs03929097_g1
*MT-ATP6*	*Mitochondrially encoded ATP synthase 6*	Component of ATP synthase complex V, ATP production via oxidative phosphorylation	Hs02596862_g1
*CDKN1A*	*cyclin-dependent kinase inhibitor 1A*	Regulatory enzyme in cell cycle progression	Hs00355782_m1

### Data analysis and statistics

The threshold cycle (Ct) value, which is inversely proportional to the target mRNA abundance, was used to estimate the level of gene expression. The inverse of the Ct value (that is, 1/Ct), therefore, gives the abundance value of the mRNA. Relative stability was determined by the ΔCt method [18], comparing all possible gene combinations. The level of variability was indicated by the range of the standard deviation of the Ct values (StdDev) across samples. In this method, comparison of the ΔCt values of the different genes provides information on which pairs show less variability and hence which genes are stably expressed among the samples tested. A relatively large panel of genes can be compared against one another and either chosen or discarded on the basis of ΔCt. The average ΔCt is derived by dividing the ΔCt of one gene with that of another, and the average standard deviation is a measure of the gene expression variability. Further data analysis was carried out using NormFinder software [32]. NormFinder provides intra- and inter-variability, the best endogenous control, and also the best combination of two endogenous controls. The NormFinder applies a mathematical model to separate the analysis of the sample subgroups, estimates both the intra- and the intergroup expression variations, and calculates the stability value of a candidate gene. It works on a Microsoft Excel platform that automatically calculates the stability value for all candidate normalization genes containing any number of samples arranged in any number of groups. This approach ranks the best candidate gene with the minimal estimated intra- and intergroup variation, whereas the pair-wise comparison approach tends to select those genes with the highest degree of similarity across the sample sets. In the pair-wise comparison approach, the gene with the minimum expression variation does not necessarily get chosen as the best candidate gene. The most stable gene expression is indicated by the lowest average expression stability value. All data are shown as mean ± SD. One-way ANOVA was employed to determine the statistical significance of Ct values. Differences of *P* < 0.05 were considered statistically significant.

## RESULTS AND DISCUSSION

Genome-wide studies have provided an insight into possible perturbations of biological functions in human peripheral blood lymphocytes (HPBLs) following γ-irradiation [33–35]. Exposure of HPBLs to environmental stresses, including ionizing radiation, is known to activate multiple signal transduction pathways, and rapidly results in complex patterns of gene expression change. As a biological material, human whole blood offers a great advantage, since circulating lymphocytes are both sensitive to early radiation injury and also highly responsive in terms of induced gene expression changes. As they are also relatively easily biopsied, non-stimulated HPBLs provide an ideal model for development of a gene expression biodosimeter for radiation exposure. qPCR is one of the most sensitive and reproducible relative quantification methods for gene expression analysis and provides simultaneous measurement of gene expression in many different samples. In qPCR, selection of an ideal housekeeping gene is an important criterion for a reliable and accurate interpretation of results. Therefore, any candidate housekeeping gene for the purpose of differential gene expression analysis should remain stably expressed between samples taken from different timepoints and under different experimental conditions [18]. The most commonly used housekeeping genes, such as *18S*, *GAPDH* and *ACTB* are known to vary considerably in their transcriptional levels between different individuals, different cell types, different developmental stages and under different experimental conditions [19, 20, 36]. Even though the level of ribosomal 18S is not a direct indicator of mRNA level or gene expression, it is also used widely in gene expression analysis.

**Fig. 1.**
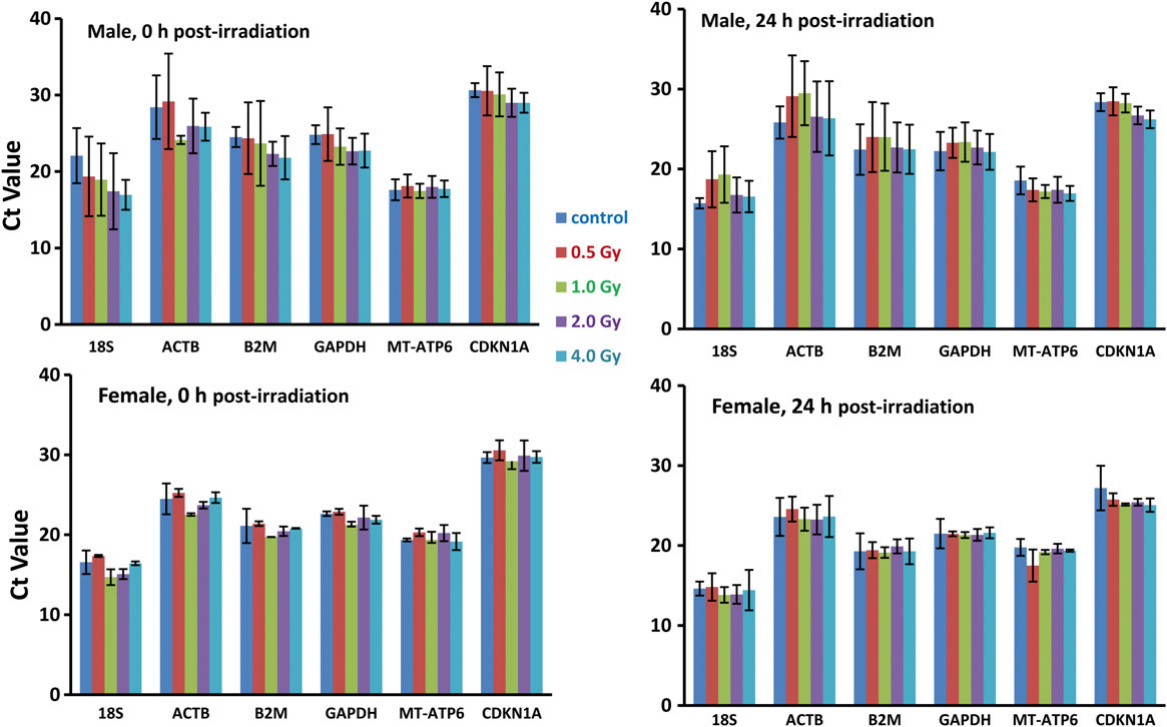
Ct values representing the expression levels of six housekeeping genes in human whole blood samples in the sham-exposed control and groups exposed to 0.5 Gy, 1 Gy, 2 Gy and 4 Gy doses of ^60^Co γ-radiation at 0 (left panels) and 24 h (right panels) post-irradiation in male (top panels) and female (bottom panels) blood samples. The bars represent the statistical means of the Ct values for different individuals within a group, and the SD represents the range of variation within a group. (Differences of *P* < 0.05 were considered statistically significant.)

**Fig. 2.**
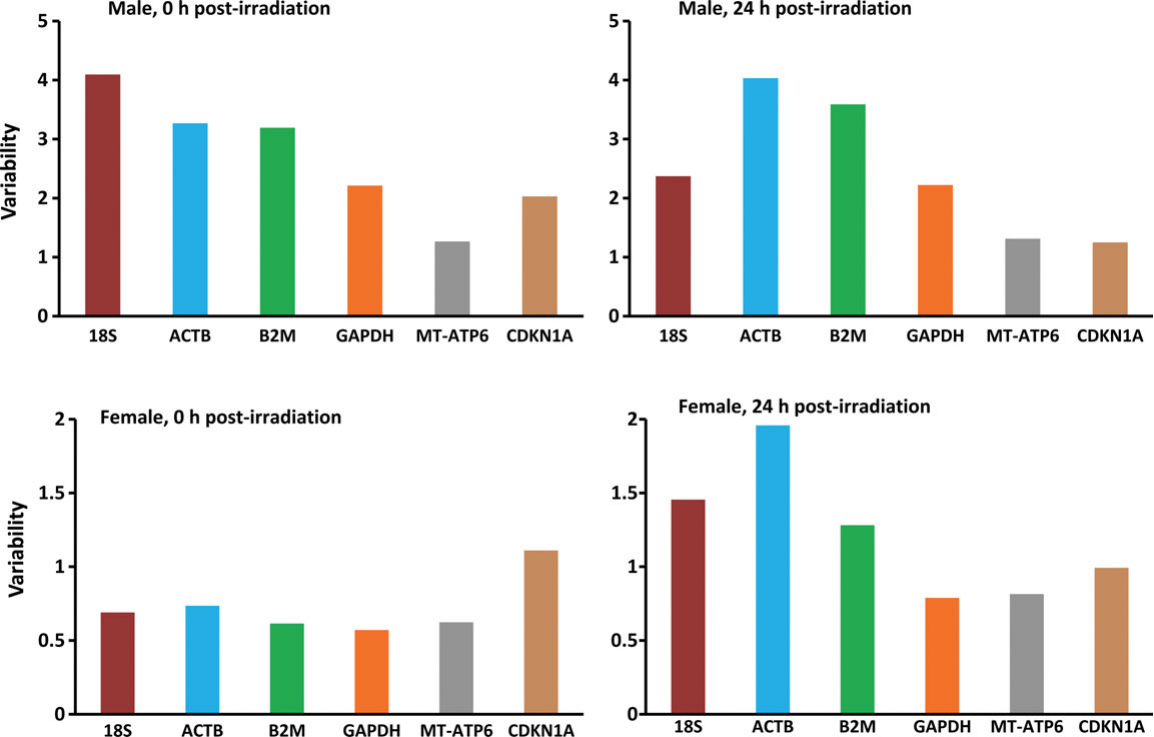
Gene variability as determined by comparison of Ct values. Variability of gene expression was estimated by comparing the standard deviations (StdDev) of the Ct values. The average StdDev represents the variation in gene expression level in the different experimental groups. (Differences of *P* < 0.05 were considered statistically significant.)

In this study, we first compared the gene expression levels of the six chosen genes by a direct Ct method, which gave some indication of the overall expression variations [25]. To analyze whether or not the gene expression was affected by γ-irradiation, the average Ct values for each group were compared (Fig. 1). The mean Ct value ranged from 15.71 to 30.65 in males (Fig. 1, top panels) and 13.83 to 30.5 in females (Fig. 1, bottom panels). It ranged between 14.7 and 30.5 at 0 h post-irradiation incubation (Fig. 1, left panels), whereas it ranged between 13.8 and 28.4 at 24 h post-irradiation incubation (Fig. 1, right panels). All six housekeeping genes exhibited essentially similar trends in both genders and post-irradiation incubation groups. We further analyzed the data to look for statistically significant difference between the two genders by one-way ANOVA between pairs of gender groups (Male Control vs Female Control; Male 0.5 Gy vs Female 0.5 Gy, etc.) The results suggest that there is no significant difference between the two (Supplementary Table 1). With the exception of *MT-ATP6* for the 1.0, 2.0 and 4.0 Gy groups, the *P* values are greater than 0.05 and hence fail to reject the null hypothesis. Similarly, comparison of the Ct values combining both the genders also suggests that there is no statistical difference between different dose groups at 0 h and 24 h post-irradiation (Supplementary Table 2). We are aware that small sample size could be a critical factor in this outcome. The order of abundance (1/Ct) of the genes covered in this study, was *18S*>*MT-ATP6*>*B2M*>*GAPDH*>*ACTB*>*CDKN1A* in all experimental groups comprising both gender and post-irradiation incubation period groups (Fig. 1). In order to determine the effect of radiation upon expression level, the Ct values of the control (or sham-exposed) samples were compared with exposed samples (Supplementary Table 3). In this case also, no significant dose effect was observed. However, different experimental groups individually exhibited intragroup variations. The standard deviation (STDev) of the average Ct values was used to represent the ‘range of variability’ of gene expression level. In males, the order of variability was *18S*>*ACTB*>*B2M*>*GAPDH*>*CDKN1A*>*MT-ATP6* at 0 h and *ACTB*>*B2M*>*18S*>*GAPDH*>*MT-ATP6*>*CDKN1A* at 24 h post-irradiation (Fig. 2, top panels), while in the case of females, the orders were *CDKN1A*>*ACTB*>*18S*>*MT-ATP6*>*B2M*>*GAPDH* at 0 h and *ACTB*>*18S*>*B2M*>*CDKN1A*>*MT-ATP6*>*GAPDH* at 24 h post-irradiation (Fig. 2, bottom panels). However, comparison of the variability of each gene across the dose range suggested that there were no significant differences in any of the groups. For instance, in the case of *18S*, there was no significant difference in the level of expression between *18S* and any other gene under comparison (Supplementary Table 4). From these results, the most abundantly expressed gene comes out to be *18S* followed by *MT-ATP6* in all the groups (Fig. 1). The minimum average Ct range was found for the *MT-ATP6* gene in males and the *GAPDH* gene in females (Fig. 2).

Gene expression stability was further evaluated by ΔCt and standard deviation (StdDev) methods by comparing all possible gene combinations [11, 18, 21]. The advantage of this approach was that it bypassed the need to accurately quantify input RNA, and instead employed ΔCt comparisons between the genes. This study involved six genes, making 30 possible gene combinations (Table 2). The increased level of the average StdDev of Ct values across the samples is indicative of the high variability and, therefore, low stability of gene expression, and vice versa. In this test, the genes that scored the highest for the requirements of being suitable endogenous controls were *GAPDH* and *MT-ATP6* (Table 2). The least value of the average standard deviation was observed when the *GAPDH* and *MT-ATP-6* genes were compared against the other five genes (0.755 and 0.781, respectively). *CDKN1A* and *B2M* demonstrated an intermediate level of variation (0.823 and 0.923, respectively), whereas *18S* and *ACTB* demonstrated higher levels of variability (Table 2). The variability ranking of all of the endogenous genes covered in this study, therefore, emerged as GAPDH>*MT-ATP6*>*CDKN1A*>*B2M*>*18S*>*ACTB*. This result showed that expression of the *GAPDH* gene, followed by the *MT-ATP6* gene, was the most stable in terms of expression across all the parameters in γ-ray-exposed HPBL samples. This also demonstrated that ionizing radiation had the least effect on these two genes, whereas the *ACTB* gene showed the maximum variation.

**Table 2.** Pair-wise comparison of six housekeeping genes

Sample	Average ΔCt	StdDev	Average StdDev
*18S* vs *ACTB*	0.885	0.918	**1**.**597**
*18 s* vs *B2M*	1.605	1.599	
*18S* vs *GAPDH*	2.086	1.880	
*18S* vs *MT-ATP6*	1.851	1.831	
*18S* vs *CDKN1A*	1.572	1.756	
*ACTB* vs *18S*	1.129	1.088	**1**.**756**
*ACTB* vs *B2M*	1.812	1.740	
*ACTB* vs *GAPDH*	2.090	2.046	
*ACTB* vs *MT-ATP6*	2.090	1.99	
*ACTB* vs *CDKN1A*	1.775	1.912	
*B2M* vs *18S*	0.622	0.625	**0**.**923**
*B2M* vs *ACTB*	0.551	0.574	
*B2M* vs *GAPDH*	1.299	1.175	
*B2M* vs *MT-ATP6*	1.153	1.145	
*B2M* vs *CDKN1A*	0.979	1.098	
*GAPDH* vs *18S*	0.479	0.531	**0**.**755**
*GAPDH* vs *ACTB*	0.424	0.488	
*GAPDH* vs *B2M*	0.769	0.850	
*GAPDH* vs *MT-ATP6*	0.887	0.973	
*GAPDH* vs *CDKN1A*	0.753	0.934	
*MT-ATP6* vs *18S*	0.539	0.545	**0**.**781**
*MT-ATP6* vs *ACTB*	0.478	0.501	
*MT-ATP6* vs *B2M*	0.867	0.873	
*MT-ATP6* vs *GAPDH*	1.126	1.026	
*MT-ATP6* vs *CDKN1A*	0.848	0.959	
*CDKN1A* vs *18S*	0.636	0.569	**0**.**823**
*CDKN1A* vs *ACTB*	0.563	0.522	
*CDKN1A* vs *B2M*	1.021	0.910	
*CDKN1A* vs *GAPDH*	1.327	1.070	
*CDKN1A* vs *CDKN1A*	1.177	1.042	

Average ΔCt values represent mean difference between the genes across 30 samples. Standard deviation (StdDev) represents variation in Ct values across the samples.

**Fig. 3.**
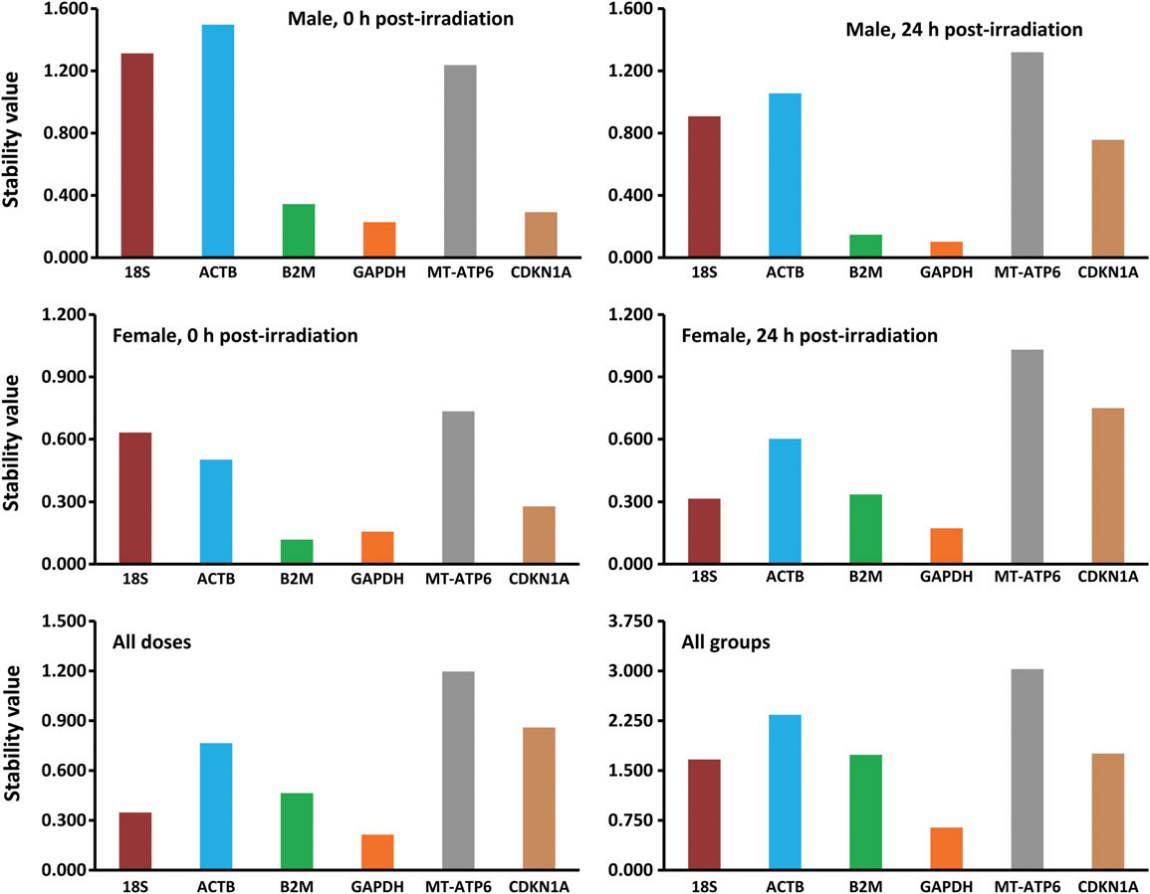
The gene stability values of six housekeeping genes, as predicted by the NormFinder algorithm for a number of experimental groups comprising both genders (top and middle panels), two post-irradiation periods (top and middle panels), and the different irradiation groups together (bottom left panel), and both genders as well as irradiation groups together (bottom right panel). The lowest stability value indicates the most stable gene and vice versa.

**Fig. 4.**
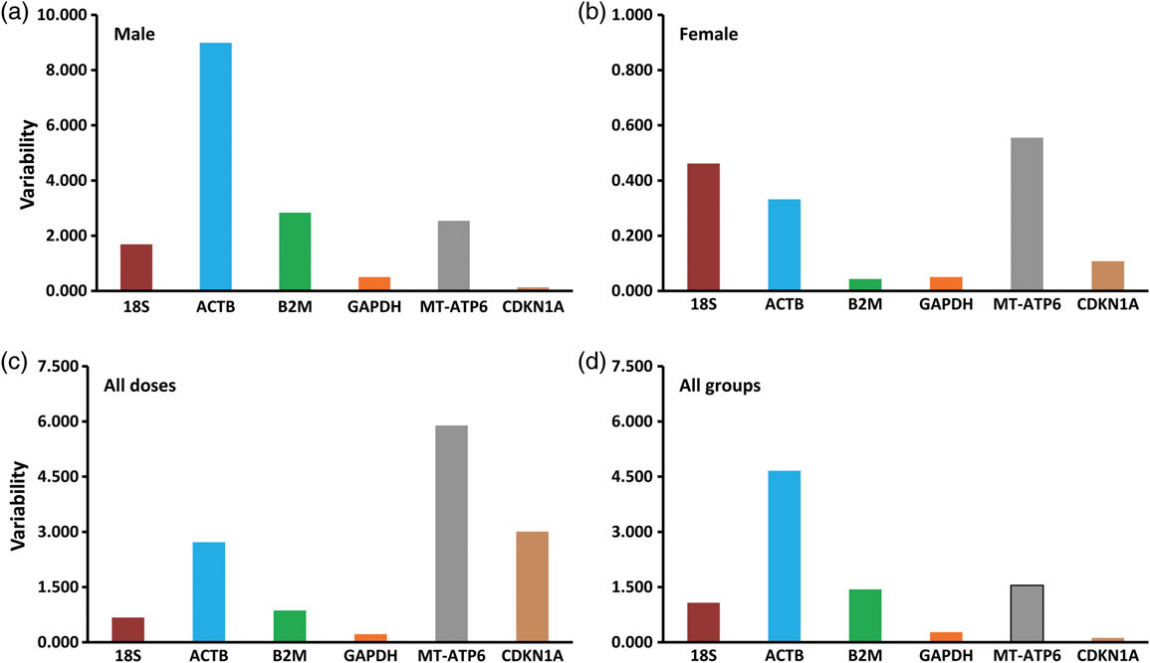
Intragroup variations for six housekeeping genes from different study groups, namely: (**a**) male, (**b**) female, (**c**) all doses and (**d**) all groups combined. The variability value of each gene represents the level of variation of a gene across the different groups.

Since all the genes selected for this study have different functions, the possibility of coregulation or coordinate expression can be ruled out. The NormFinder algorithm, being rooted in a mathematical gene expression model, employs a solid statistical framework for estimating the variation between sample subgroups within a sample set [18, 26, 32]. In the earlier Ct approach, we could only estimate the overall gene expression variation, without taking into account the systematic intergroup variation, which is critical in correct interpretation of results [25, 37]. NormFinder can discriminate between different groups based on a given group identifier (e.g. 0 Gy, 1 Gy, 2 Gy and 4 Gy samples) and combines both intra- and inter-group variations into a stability value for each gene [14]. The gene with the lowest stability value signifies the most stable gene within the groups under investigation. Besides, it also suggests the best combinations of two genes within a group. The NormFinder algorithm ranks the six genes from irradiated HPBLs based on their expression stability, as shown in Fig. 3. Overall, the *GAPDH* gene was the most stably expressed gene with the lowest stability value, closely followed by the *B2M* and *18S* genes (Table 3). The intragroup variations were also estimated by the NormFinder for each of the experimental groups (Fig. 4). In males, the *CDKN1A* and *GAPDH* genes were the least variable, whereas in females, the least variable were the *B2M* and *GAPDH* genes (Fig. 4, top panels). In the different dosage groups, *GAPDH* showed least variation, followed by *18S* and *B2M* (Fig. 4, bottom left). When all experimental groups were combined, *CDKN1A* and *GAPDH* showed the least variation, followed by *18S* (Fig. 4, bottom right). The best combination of two genes was also predicted by the NormFinder program for each experimental group (Table 3), with the best combination represented by the lowest stability values. The *GAPDH* and *18S* genes, by far, appear to be the best combination of two genes to serve as the endogenous control under the experimental conditions employed in our study. The variability observed in the case of the *β-actin* and *CDKN1A* genes can be explained by earlier findings that showed the effect of radiation on the expression levels of these genes [22, 28].

**Table 3.** Best combination of two genes predicted by NormFinder

	Male	Female	Different dosage	All doses and genders
**Gene Combination**	GAPDH MT-ATP6	B2M 18S	GAPDH 18S	GAPDH 18S
**Stability Value**	0.63	0.41	0.2	0.8

## CONCLUSION

The results we obtained using a number of different approaches are essentially similar, suggesting that *GAPDH* is the most stable and abundant endogenous control gene, closely followed by the *18S* gene. Therefore, from this study, we proposed that gene expression analysis involving qPCR of human whole blood exposed to ionizing radiation, such as ^60^Co γ-rays, should employ either the *GAPDH* gene alone or in combination with the *18S* gene as the endogenous control for the most accurate and reliable interpretation of results. We do not rule out use of these endogenous controls in other gene expression studies involving interventions other than radiation.

## SUPPLEMENTARY DATA

Supplementary data is available at the *Journal of Radiation Research* online.

## FUNDING

The authors thank the funding agencies Board of Research in Nuclear Sciences (BRNS), (Department of Atomic Energy, India), Science and Engineering Research Board (SERB) (Department of Science and Technology, India) and International Atomic Energy Agency (IAEA) (Vienna, Austria) (Grant # 17047) for their shared financial support of this investigation.

## Supplementary Material

Supplementary Data
