# Supplementary material for: Evaluation of endogenous control gene(s) for gene expression studies in human blood exposed to 60Co γ-rays ex vivo
Source: J Radiat Res. 2014 Sep 30;56(1):177–85. doi: 10.1093/jrr/rru074 (PMC4572586; doi:10.1093/jrr/rru074)
Supplement: Supplementary Data [file supp_56_1_177__index.html]

Evaluation of endogenous control gene(s) for gene expression studies in human blood exposed to 60Co γ-rays ex vivo — Supplementary Data 

# Evaluation of endogenous control gene(s) for gene expression studies in human blood exposed to 60Co γ-rays *ex vivo*

## Supplementary Data

Supplementary Data

**Files in this Data Supplement:**

- Supplementary Data - Doc file
