# Supplementary material for: Evaluation of endogenous control gene(s) for gene expression studies in human blood exposed to 60Co γ-rays ex vivo
Source: J Radiat Res. 2014 Sep 30;56(1):177–85. doi: 10.1093/jrr/rru074 (PMC4572586; doi:10.1093/jrr/rru074)
Supplement: Supplementary Data [file supp_rru074_rru074supp_data.doc]

| **Male Vs Female** | ***p-value*** | | | | | |
| --- | --- | --- | --- | --- | --- | --- |
|  | **18S** | **ACTB** | **B2M** | **GAPDH** | **MT-ATP6** | **CDKN1A** |
| Male Con vs Female Con | 0.175 | 0.128 | 0.06 | 0.27 | 0.102 | 0.385 |
| Male 0.5 vs Female 0.5 Gy | 0.207 | 0.145 | 0.115 | 0.21 | 0.309 | 0.462 |
| Male 1.0 vs Female 1.0 Gy | 0.036 | 0.089 | 0.086 | 0.105 | 0.001 | 0.215 |
| Male 2.0 vs Female 2.0 Gy | 0.189 | 0.18 | 0.081 | 0.371 | 0.021 | 0.904 |
| Male 4.0 vs Female 4.0 Gy | 0.29 | 0.293 | 0.187 | 0.524 | 0.01 | 0.89 |

| **Post-irradiation**  **period** | ***p-value*** | | | | | |
| --- | --- | --- | --- | --- | --- | --- |
|  | **18S** | **ACTB** | **B2M** | **GAPDH** | **MT-ATP6** | **CDKN1A** |
| 0 h | 0.548 | 0.302 | 0.812 | 0.425 | 0.864 | 0.725 |
| 24 h | 0.716 | 0.789 | 0.982 | 0.962 | 0.532 | 0.243 |

**Supplementary Table 2:** Statistical analysis to determine dose effect at 0 h and 24 post-irradiation period using both gender groups. (Differences of *p*<0.05 were considered statistically significant)

**Supplementary Table 1:** Statistical analysis between male and female groups using one-way ANOVA. (Differences of *p*<0.05 were considered statistically significant)

**SUPPLEMENTARY TABLES**

| **0 h** | ***p-value*** | | | | | |
| --- | --- | --- | --- | --- | --- | --- |
|  | **18S** | **ACTB** | **B2M** | **GAPDH** | **MT-ATP6** | **CDKN1A** |
| Control Vs 0.5 Gy | 0.612 | 0.79 | 0.992 | 0.921 | 0.493 | 0.791 |
| Control Vs 1.0 Gy | 0.336 | 0.09 | 0.653 | 0.22 | 0.927 | 0.633 |
| Control Vs 2.0 Gy | 0.206 | 0.42 | 0.24 | 0.15 | 0.569 | 0.325 |
| Control Vs 4.0 Gy | 0.139 | 0.44 | 0.246 | 0.158 | 0.998 | 0.164 |

| **24 h** | ***p-value*** | | | | | |
| --- | --- | --- | --- | --- | --- | --- |
|  | **18S** | **ACTB** | **B2M** | **GAPDH** | **MT-ATP6** | **CDKN1A** |
| Control Vs 0.5 Gy | 0.264 | 0.319 | 0.669 | 0.612 | 0.118 | 0.673 |
| Control Vs 1.0 Gy | 0.336 | 0.377 | 0.707 | 0.65 | 0.256 | 0.454 |
| Control Vs 2.0 Gy | 0.77 | 0.879 | 0.831 | 0.864 | 0.472 | 0.097 |
| Control Vs 4.0 Gy | 0.696 | 0.871 | 0.992 | 0.986 | 0.262 | 0.046 |

|  | ***p-value*** |
| --- | --- |
| **18S vs ACTB** | 0.749 |
| **18S vs B2M** | 0.987 |
| **18S vs GAPDH** | 0.443 |
| **18S vs MT-ATP6** | 0.177 |
| **18S vs CDKN1A** | 0.334 |

**Supplementary Table 3:** Statistical analysis comparing between control and irradiated samples at 0 h and 24 h post-irradiation time intervals. Differences of *p*<0.05 were considered statistically significant)

**Supplementary Table 4 :** Statistical analysis with one-way ANOVA for comparison between 18S and other endogenous genes across different dose groups. (Differences of *p*<0.05 were considered statistically significant).
